# Tracking Biodistribution of Myeloid-Derived Cells in Murine Models of Breast Cancer

**DOI:** 10.3390/genes10040297

**Published:** 2019-04-12

**Authors:** Jun Li, Junhua Mai, Louis Hinkle, Daniel Lin, Jingxin Zhang, Xiaoling Liu, Maricela R. Ramirez, Youli Zu, Ganesh L. Lokesh, David E. Volk, Haifa Shen

**Affiliations:** 1 Department of Nanomedicine, Houston Methodist Hospital Research Institute, Houston, TX 77030, USA; jli6@houstonmethodist.org (J.L.); jmai@houstonmethodist.org (J.M.); lehinkle@houstonmethodist.org (L.H.); dlin@houstonmethodist.org (D.L.); jzhang4@houstonmethodist.org (J.Z.); liuxiaoling97@aliyun.com (X.L.); MReyes2@houstonmethodist.org (M.R.R.); 2 Xiangya School of Medicine, Central South University, 410008 Changsha, Hunan, China; 3 Department of Pathology and Genomic Medicine, Houston, TX 77030, USA; yzu@houstonmethodist.org; 4 Institute of Molecular Medicine, McGovern Medical School, The University of Texas Health Science Center at Houston, Houston, TX 77030, USA; loks154@yahoo.com (G.L.L.); David.Volk@uth.tmc.edu (D.E.V.); 5 Cancer Center, Houston Methodist Hospital, Houston, TX 77030, USA; 6 Department of Cell and Developmental Biology, Weill Cornell Medicine, New York, NY 10065, USA

**Keywords:** breast cancer, myeloid-derived suppressor cell, biodistribution, thioaptamer

## Abstract

A growing tumor is constantly secreting inflammatory chemokines and cytokines that induce release of immature myeloid cells, including myeloid-derived suppressor cells (MDSCs) and macrophages, from the bone marrow. These cells not only promote tumor growth, but also prepare distant organs for tumor metastasis. On the other hand, the myeloid-derived cells also have phagocytic potential, and can serve as vehicles for drug delivery. We have previously identified thioaptamers that bind a subset of MDSCs with high affinity and specificity. In the current study, we applied one of the thioaptamers as a probe to track myeloid cell distribution in the bone, liver, spleen and tumor in multiple murine models of breast cancer including the 4T1 syngeneic model and MDA-MB-231 and SUM159 xenograft models. Information generated from this study will facilitate further understanding of tumor growth and metastasis, and predict biodistribution patterns of cell-mediated drug delivery.

## 1. Introduction

A growing tumor is increasingly infiltrated with immature myeloid-derived suppressor cells (MDSCs) comprising polymorphonuclear MDSCs (PMN-MDSCs) and monocytic MDSCs (M-MDSCs), the latter can differentiate into tumor-associated macrophages (TAMs) in the inflammatory tissue [1,2]. These tumor-associated myeloid cells produce high levels of reactive oxygen and nitrogen species, and anti-inflammatory cytokines, and are thus responsible for suppression of anti-tumor functions of other immune cells, including T and B lymphocytes and natural killer cells [3,4,5,6]. In the meantime, the tumor infiltration properties of myeloid-derived cells also provide an avenue for delivery of cancer therapeutic agents using the cells as vehicles [7]. Thus, it is important to precisely track migration of the myeloid cells and to understand their biodistribution pattern in tumor tissues in the major organs. It is also essential to identify probes/ligands that can bind the tumor-infiltrating cells will high affinity and specificity.

Nucleic acid-based aptamers are single-stranded oligonucleotides with unique secondary structures. Using an in vivo systematic evolution of ligands by the exponential enrichment (SELEX) approach [8,9,10], we have previously screened a thioaptamer library and identified a group of aptamers that were enriched in the tumor tissues in murine models of primary breast cancer [11,12]. One of the aptamers, the T1 aptamer, showed high binding affinity to the granulocytes/PMN-MDSCs and a selected number of tumor cells. It was used as an affinity moiety for targeted drug delivery to primary breast cancer [11]. Another aptamer also demonstrated high affinity to human lymphoma, and served as an affinity moiety for drug delivery to lymphoma [12].

Here in our study, we tested the T1 binding affinity to granulocytes and macrophages in three different breast cancer models. Our results showed that the T1 aptamer was able to recognize and bind onto granulocytes and some macrophages in different breast cancer models. Moreover, this T1 aptamer has shown a strong binding ability onto human bone marrow hematopoietic cells. These are promising results for when we target granulocytes and aim to rescue anti-tumor immunity in the tumor microenvironment. Not restricted to murine cancer models, the T1 aptamer is also a promising tool to target human cells, where there is a strong indication that the T1 aptamer can be applied in clinical use.

## 2. Materials and Methods

### 2.1. Antibodies and Aptamers

Anti-mouse CD45, Ly6G, Ly6C and F4/80 antibodies were purchased from ThermoFisher (Waltham, MA, USA). Anti-CD11b antibody was from Tonbo Biosciences (San Diego, CA, USA). Anti-human CD45 antibody was from BD Bioscience (San Jose, CA, USA). Cy5 fluorescent dye-conjugated T1 and scramble aptamers were ordered from Integrated DNA Technologies (Coralville, IA, USA).

### 2.2. Cell Culture

The 4T1 and MDA-MB-231 cancer cell lines were purchased from American Type Culture Collection (Manassas, VA, ATCC), SUM159 was from Asterand (Detroit, MI, USA). Cells were cultured in Dulbecco’s Modified Eagle’s Medium (DMEM, Corning, NY, USA) supplemented with 10% fetal bovine serum (FBS, ThermoFisher), penicillin (100 IU/mL) and streptomycin (100 μg/mL) (Cellgro, Corning, NY, USA) at 37 °C with 5% CO_2_.

### 2.3. Murine Tumor Models

All animal studies were performed following protocols approved by the Institutional Animal Care and Use Committee (IACUC) at the Houston Methodist Research Institute. Female athymic nude mice and BALB/c mice (6–10 weeks old) were purchased from Charles River Laboratories (Boston, MA, USA). Human xenograft tumor models were generated by inoculating 3 × 10^6^ MDA-MB-231 or SUM159 cells in the mammary gland fat pads of female athymic nude mice, and 4T1 syngeneic tumor models were established by inoculating 1 × 10^6^ 4T1 cells into mammary gland fat pads of female BALB/c mice.

### 2.4. T1 Inoculation and Sample Preparation

Mice were administered 0.5 nmol Cy5-SCR or Cy5-T1 aptamer per mouse via a tail vein injection. Bone, liver, spleen and tumor were collected four hours later. Single cell suspensions from spleens were prepared by grinding the tissue through 40-μm nylon filters (BD Biosciences, San Jose, CA, USA), and bone marrow cells were harvested from the femur and tibia by flushing them with phosphate buffer saline (PBS). To prepare single cells from tumor and liver, the tissues were minced with scalpels and incubated with 250 U/mL collagenase type III (Worthington Biochem, Lakewood, NJ, USA) for 1 h at 37 °C. The samples were then filtered through 70-μm nylon filters (BD Biosciences, San Jose, CA, USA), and lysed with ACK lysing buffer (Lonza, Alpharetta, GA, USA). Single cells were then washed with PBS and resuspended in 2% FBS.

### 2.5. Flow Cytometry Analysis

Cells were stained with antibodies for 30 min at 4 °C before they were applied for flow cytometry analysis. The CD45^+^ cells were grouped based on the following surface markers: CD11b^+^Ly6G^+^Ly6C^low^ for granulocytes, CD11b^+^Ly6G^−^Ly6C^high^ for M-MDSCs, and CD11b^+^F4/80^+^ for macrophages.

### 2.6. Aptamer to Human Bone Marrow Hematopoietic Cells

T1 and scramble aptamers were diluted with a binding buffer containing 1 mg/mL bovine serum albumin (BSA), 4.5 mg/mL glucose, 0.1 mg/mL yeast t-RNA, and 5 mM MgCl_2_ in PBS. Human bone marrow hematopoietic cells from a healthy donor were incubated with increasing concentrations (1.56 nM, 3.12 nM, 6.25 nM, 12.5 nM, and 25 nM) of T1 or scramble aptamer and anti-human CD45 antibody for 20 min before they were applied for flow cytometry analysis. Human hematopoietic cells (CD45^+^) were gated for assessment of aptamer binding.

### 2.7. Statistical Analysis

Statistical analysis was performed with the GraphPad Prism 5 program (GraphPad Software, Inc, CA, USA). Comparison between two groups was evaluated with a two-tailed Student’s *t*-test. Data is presented as mean ± s.d. (*n* = 4). *p* values are from a two-tailed student *t*-test. *, *p* < 0.05; **, *p* < 0.01; ***, *p* < 0.001; ****, *p* < 0.0001; *****, *p* < 0.00001; ns, not significant.

## 3. Results

### 3.1. The T1 Aptamer Binds Granulocytes and Macrophages in Murine Orthotopic Breast Cancer Model

**Figure 1:**
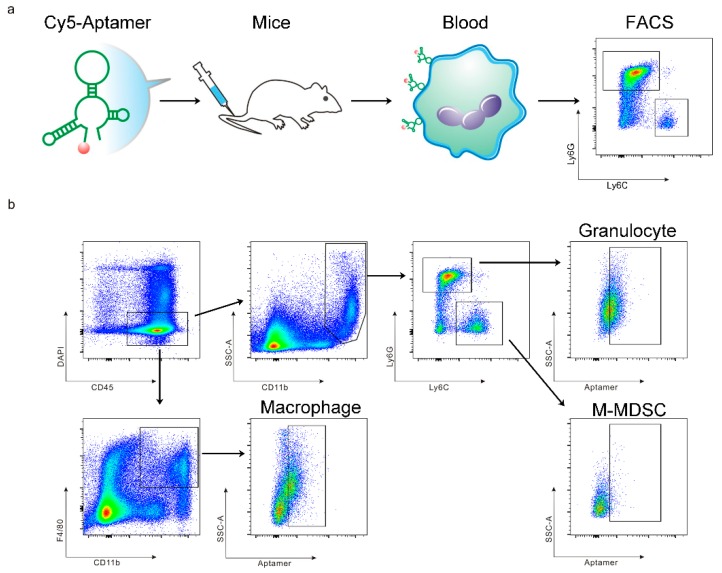
Schematic view on study design and myeloid cell separation. (**a**) Schematic view of research procedure. (**b**) Gating strategy for detection of aptamer-positive CD45^+^CD11b^+^Ly6G^+^Ly6C^low^ granulocytes, CD45^+^CD11b^+^Ly6G^−^Ly6C^high^ M-MDSCs, and CD45^+^CD11b^+^F4/80^+^ macrophages.

**Figure 2:**
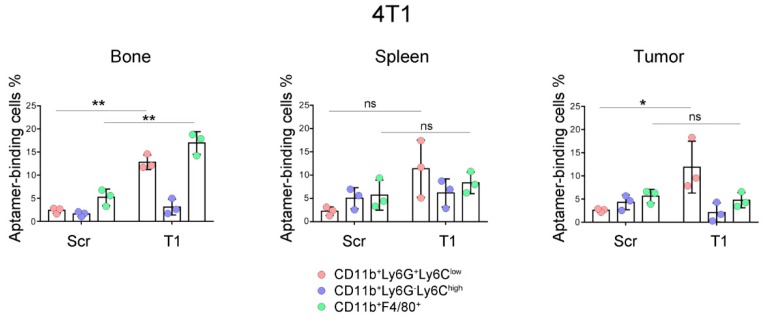
Analysis of aptamer-binding myeloid cells in murine model of primary 4T1 mammary gland tumor. Mice bearing primary 4T1 tumors (*n* = 3 mice/group) were treated with Cy5-labeled T1 or scramble aptamer. They were euthanized 4 h later, and single cells were prepared from bone marrow, spleen and tumor. Flow cytometry was performed to detect aptamer-positive cells in the granulocytes, M-MDSC and macrophage populations. Pink: CD11b^+^Ly6C^low^Ly6G^+^ granulocytes; Blue: CD11b^+^Ly6C^high^Ly6G^−^ M-MDSCs; Green: CD11b^+^F4/80^+^ macrophages. Data is presented as mean ± s.d. *P* values are calculated with a two-tailed student *t*-test. *, *p* < 0.05; **, *p* < 0.01; ns, not significant.

In a previous study, we demonstrated that the T1 aptamer could bind the CD11b^+^Ly6G^+^ myeloid cells with specificity in BALB/c mice bearing primary 4T1 mammary gland tumors [11]. As the sub-populations of myeloid cells carry their specific surface markers, we performed further analysis in this study to determine T1 binding to the CD11b^+^Ly6G^+^Ly6C^low^ granulocytes and CD11b^+^Ly6G^−^Ly6C^high^ M-MDSCs. In addition, we measured T1 binding to the CD11b^+^F4/80^+^ macrophages. These 3 types of myeloid-derived cells could be easily separated with flow cytometry (Figure 1). Interestingly, we detected different T1 binding patterns in cells from different organs. Elevated T1 binding was detected in both granulocytes and macrophages from the bone marrow samples, while only the granulocytes showed elevated T1 binding in the tumor samples (Figure 2). The splenic samples showed the same trend as the tumor samples, although statistical significance was not detected. As expected, there was no detectable T1 binding to the M-MDSCs in samples from all three tissue types. The results indicate that the T1 aptamer can serve as a unique probe to measure levels of granulocytes and macrophages in both the tumor tissue and other major organs.

### 3.2. T1 Aptamer Binds Myeloid Cells in Murine Models of Xenograft Tumors

**Figure 3:**
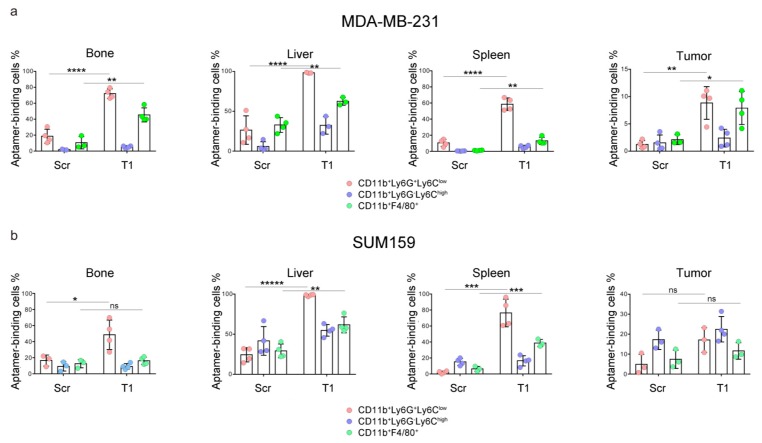
Analysis of aptamer-binding myeloid cells in a murine model of MDA-MB-231 and SUM159 xenograft tumors. Mice bearing primary (**a**) MDA-MB-231 or (**b**) SUM159 primary tumors (*n* = 4 mice/group) were treated with Cy5-labeled T1 or scramble aptamer. They were euthanized 4 h later, and single cells were prepared from bone marrow, liver, spleen and tumor tissues. Flow cytometry was performed to detect aptamer-positive cells. Pink: CD11b^+^Ly6C^low^Ly6G^+^ granulocytes; Blue: CD11b^+^Ly6C^high^Ly6G^−^ M-MDSCs; Green: CD11b^+^F4/80^+^ macrophages. Data is presented as mean ± s.d. *p* values are calculated with a two-tailed student *t*-test. *, *p* < 0.05; **, *p* < 0.01; ***, *p* < 0.001; ****, *p* < 0.0001; *****, *p* < 0.00001; ns, not significant.

We expanded the study to athymic nude mice carrying MDA-MB-231 and SUM159 xenograft human breast cancers, and analyzed T1 aptamer binding to myeloid cells in the bone marrow, liver, spleen and tumor. Both tumor lines are well characterized and have been applied in our previous studies [13,14,15,16]. As bone and liver are common metastatic organs for breast cancer [17], accumulation of the immunosuppressive myeloid cells in these organs may facilitate cancer metastasis. As expected, we detected high levels of T1 binding to the granulocytes in the bone marrow, livers and spleens in mice with primary MDA-MB-231 tumors (Figure 3). A 5-fold increase of T1 binding to tumor-derived granulocytes was also detected, although overall percentage of T1-positive cells in the tumor was much lower than that in the non-tumor tissues. As in mice with 4T1 tumors, significant T1 binding to macrophages in bone marrow was detected. A similar pattern of T1 binding was also observed in liver-associated macrophages. In contrast to the 4T1 tumors, higher T1 binding to the tumor-associated macrophages was detected.

A different T1-binding pattern was observed in samples isolated from mice with a SUM159 tumor, another line of human triple negative breast cancer (Figure 3). While consistent T1 binding to the granulocytes was observed, elevated T1 binding to macrophages was detected in the liver and spleen samples. These studies demonstrate universal application potential of the T1 aptamer as a probe for immature myeloid cells.

### 3.3. T1 Aptamer Preferentially Binds on Human Bone Marrow Hematopoietic Cells

**Figure 4:**
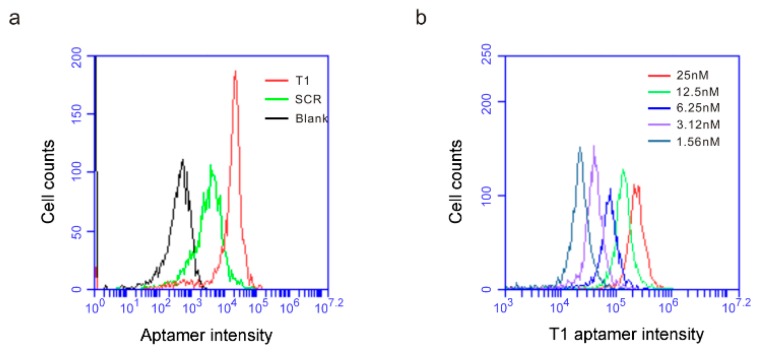
T1 aptamer preferentially binds human bone marrow cells. (**a**) Human bone marrow cells were incubated with increasing concentrations of Cy5-labeled T1 or SCR aptamers, and flow cytometry was performed to detect aptamer-binding cells. (**a**) Separation of scramble- and T1 aptamer-binding human bone marrow hematopoietic cells. Aptamer concentration: 1.56 nM. (**b**) T1 aptamer concentration-dependent binding of human bone marrow hematopoietic cells.

Bone marrow is the primary source of immature myeloid cells. In an effort to examine whether T1 aptamer was applicable to human cells, we incubated Cy5-labeled T1 and scramble aptamers with human CD45^+^ bone marrow hematopoietic cells and measured their cell-binding activity. At 1.56 nM concentration, the T1 aptamer displayed one magnitude higher binding affinity to the cells than the scramble aptamer (Figure 4a). In addition, T1 binding to the human bone marrow hematopoietic cells was dose-dependent (Figure 4b). The results imply that information from the murine tumor model-based studies can be applicable to human pathology. 

## 4. Discussion

The myeloid-derived cells are comprised of multiple sub-populations of mature and immature cells. In this study, we have shown that the T1 aptamer consistently binds the CD11b^+^Ly6G^+^Ly6C^low^ granulocytic sub-population of cells, but not the CD11b^+^Ly6G^−^Ly6C^high^ monocytic sub-population, in both tumor and non-tumor tissues. We have also detected T1 binding to the CD11b^+^F4/80^+^ macrophages, although binding levels vary depending on the tumor model and sample source. Regardless of the tumor model, our study has provided strong support for developing T1 as a unique probe to monitor tumor growth and metastasis.

We have recently shown that T1 aptamer can serve as a unique probe for tumor-targeted delivery of cancer therapeutic agents [11]. Treatment of 4T1 tumor-bearing mice with T1-conjugated liposomal doxorubicin (T1-Dox) dramatically inhibited tumor growth without causing severe toxicity. As there are a large number of CD11b^+^Ly6G^+^Ly6C^low^ granulocytic cells in circulation, the T1-Dox particles can be taken up by these cells and transported to the tumor tissue during cell infiltration. Alternatively, T1-Dox particles are carried over to the tumor tissue where they are internalized by the tumor associated granulocytic cells and macrophages. Results from the current study provide further support for development of T1 aptamer-conjugated cancer therapeutics.

An interesting observation from the study is that T1 aptamer can only bind a fraction of the CD11b^+^Ly6G^+^Ly6C^low^ granulocytes in most tissues. One possibility is poor T1 aptamer penetration into the tissues. It has been well documented that sequential physical and biological barriers exist inside the body that can block transport of nutrients, metabolites, diagnostic probes, and big and small molecule drugs [18]. The T1 aptamer falls into this category. Alternatively, T1 might preferentially bind a subset of the CD11b^+^Ly6G^+^Ly6C^low^ granulocytes. Although the body is filled with immature PMN-MDSCs during tumor growth, there are also a large number of mature granulocytes in circulation and in the inflammatory tissues. It is very difficult, if not impossible, to separate the mature granulocytes from the immature PMN-MDSCs based on flow cytometry, as both populations of cells carry an almost identical set of cell surface markers [1]. Future studies are needed to further understand the mechanism of the T1 aptamer–myeloid cell interaction.
